# Genomic Prediction Using LD-Based Haplotypes in Combined Pig Populations

**DOI:** 10.3389/fgene.2022.843300

**Published:** 2022-06-09

**Authors:** Haoqiang Ye, Zipeng Zhang, Duanyang Ren, Xiaodian Cai, Qianghui Zhu, Xiangdong Ding, Hao Zhang, Zhe Zhang, Jiaqi Li

**Affiliations:** ^1^ Guangdong Provincial Key Laboratory of Agro-Animal Genomics and Molecular Breeding, National Engineering Research Centre for Breeding Swine Industry, College of Animal Science, South China Agricultural University, Guangzhou, China; ^2^ Key Laboratory of Animal Genetics and Breeding of Ministry of Agriculture and Rural Affairs, National Engineering Laboratory of Animal Breeding, College of Animal Science and Technology, China Agricultural University, Beijing, China

**Keywords:** genomic prediction, whole-genome sequencing, haplotype, combined populations, linkage disequilibrium

## Abstract

The size of reference population is an important factor affecting genomic prediction. Thus, combining different populations in genomic prediction is an attractive way to improve prediction ability. However, combining multireference population roughly cannot increase the prediction accuracy as well as expected in pig. This may be due to different linkage disequilibrium (LD) pattern differences between population. In this study, we used the imputed whole-genome sequencing (WGS) data to construct LD-based haplotypes for genomic prediction in combined population to explore the impact of different single-nucleotide polymorphism (SNP) densities, variant representation (SNPs or haplotype alleles), and reference population size on the prediction accuracy for reproduction traits. Our results showed that genomic best linear unbiased prediction (GBLUP) using the WGS data can improve prediction accuracy in multi-population but not within-population. Not only the genomic prediction accuracy of the haplotype method using 80 K chip data in multi-population but also GBLUP for the multi-population (3.4–5.9%) was higher than that within-population (1.2–4.3%). More importantly, we have found that using the haplotype method based on the WGS data in multi-population has better genomic prediction performance, and our results showed that building haploblock in this scenario based on low LD threshold (*r* ^*2*^ = 0.2–0.3) produced an optimal set of variables for reproduction traits in Yorkshire pig population. Our results suggested that whether the use of the haplotype method based on the chip data or GBLUP (individual SNP method) based on the WGS data were beneficial for genomic prediction in multi-population, while simultaneously combining the haplotype method and WGS data was a better strategy for multi-population genomic evaluation.

## 1 Introduction

Genomic selection (GS), proposed by Meuwissen et al. (2001), uses single nucleotide polymorphism (SNP) to estimate the breeding values in younger individuals, which is with higher accuracy than pedigree-based parent average for many economically valuable traits. At present, GS has been widely applied in animal and plant breeding that has the advantages of decreasing the generation interval and accelerating the genetic progress (Spelman et al., 2013; Desta and Ortiz, 2014).

The size of the reference population is an important factor affecting GS. Generally, as the number of animals in the reference population increases, the accuracy of GS also increases (Meuwissen et al., 2001; Vanraden et al., 2009; Lund et al., 2011). For a small reference population, some studies have proposed to apply GS by combining multiple populations (Hayes et al., 2009; Brendum et al., 2011; Pryce et al., 2011). However, by simply combining the population, the accuracy of GS was limited or even slightly decreased (Erbe et al., 2012; Song et al., 2017; Song et al., 2019), which was probably due to the different linkage disequilibrium (LD) pattern differences between the population (Lei et al., 2013). Therefore, the accuracy of GS for multi-population can be improved by considering the LD consistency fragments across the genome between multi-population.

Some studies proposed to construct haplotypes as explanatory variables for GS (Edriss et al., 2013; Cuyabano et al., 2014; Meuwissen et al., 2014). A haplotype block (haploblock) is a region defined in the genome that consists of a set of neighboring SNPs that are more likely to be inherited together. Compared with the individual SNP markers, one main potential advantage of haploblocks is that each haploblock may be in higher LD than any individual nearby SNP with the causative mutations (Jonas et al., 2016; Hess et al., 2017). Therefore, the construction of haplotypes for GS can make up for the deficiency of multi-population GS, thus theoretically improve the accuracy of multi-population GS. In addition, constructing haplotypes to fit as covariates rather than individuals SNP could increase the prediction accuracy by improving the ability to capture short-range epistatic effects (Yong et al., 2018).

The number of SNP markers a haploblock should contain and for which regions of the genome the haploblocks should be defined are needed to be considered when building haplotype blocks. Some methods to define haploblock for GS are simply setting windows with a fixed number of contiguous SNPs (Hayes et al., 2007), a fixed range of base pairs on the genome (Hess et al., 2017; Sharifi et al., 2021), and a fixed-length in centimorgans (Sun et al., 2015), collectively termed as fixed-length haploblocks, which are in equal sizes of segments in the genome. Some complicated methods to define haploblock for GS attempt to incorporate the LD pattern across the genome (Cuyabano et al., 2015a; Won et al., 2021), for example, setting minimum pairwise LD cutoffs to group SNPs into haplotypes, termed variable-length haploblocks, which are in unequal sizes of the segments in the genome and may result in less explanatory variables than fixed-length haploblocks (Cuyabano et al., 2014). In theory, the variable-length haploblocks are more advantageous for GS than fixed-length haploblocks because the variable-length haploblocks group SNPs that are most likely to be inherited together across the genome.

However, the methods that have been proposed to construct haplotypes are based on low-density or high-density SNP panels, while the research on constructing haplotypes based on whole-genome sequencing (WGS) markers has rarely been proposed yet. The accuracy of genomic prediction was expected to increase by using the WGS data, which can provide more potential causative polymorphisms compared to the chip data (Meuwissen and Goddard, 2010; Druet et al., 2014; Hayes et al., 2014). In addition, a previous study suggested that fitting explanatory variables for haplotype alleles based on the WGS data may play an important role in the genomic prediction (Hess et al., 2017). Therefore, it is interesting to evaluate the accuracy of genomic prediction using the haplotype method based on WGS data.

The objective of this study was to evaluate the performance of multi-population GS, so as to explore the impact of SNP densities, variants representation (SNPs or haplotype alleles), and reference population size on the prediction accuracy. In our knowledge, this is the first study to construct haplotypes based on LD for multi-population GS at the sequence data level. When assessing the accuracy of GS, the training populations consist of two Yorkshire pig populations with different genetic backgrounds, and we separately validate each population, which was not included in the training populations.

## 2 Materials and Methods

### 2.1 Population and Phenotypes

**FIGURE 1:**
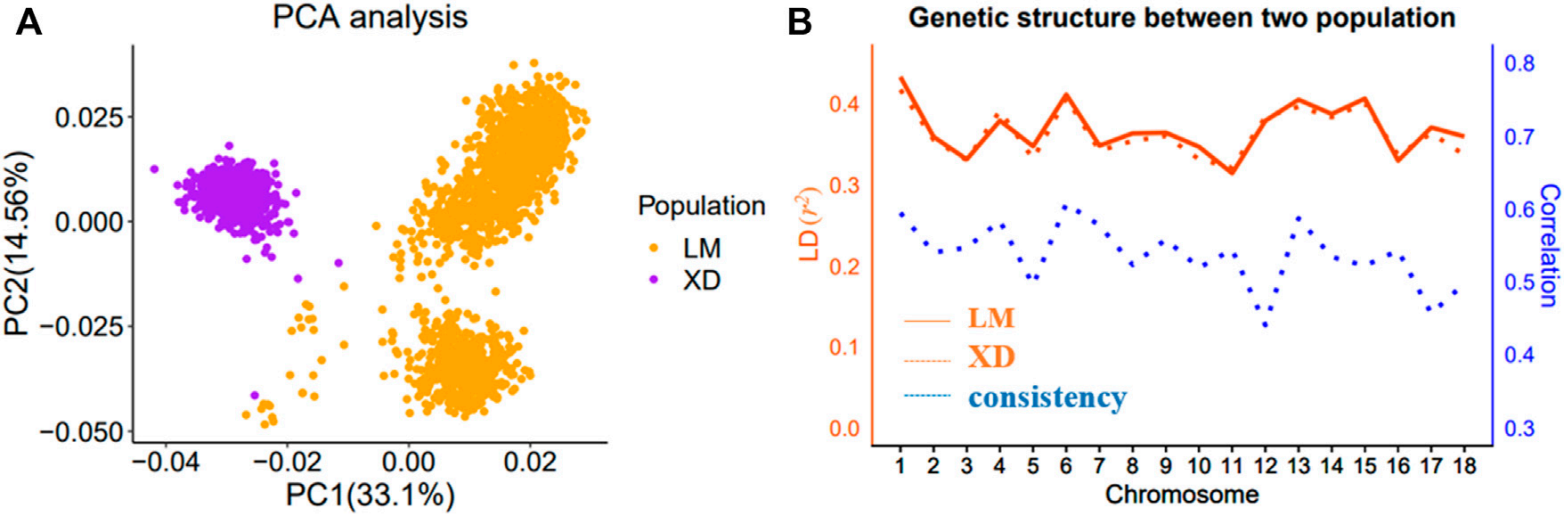
Population analysis between LM and XD. **(A)** Principal component analysis (PCA) for two population. **(B)** Genetic structure analysis for two population.

The multi-population consists of two Yorkshire pig populations, termed as LM and XD, which were the progeny of American Yorkshire and British Yorkshire pigs, respectively, and sampled from two breeding farms in China. In the LM population, there were 5,907 sows, in which 1,641 were genotyped. In the XD population, there were 4,842 sows, in which 762 were genotyped. Through the principal component analysis (PCA), we found that population structure of the two pig population was different, and there was obvious stratification of population structures (Figure 1A). The *r* ^*2*^ value (a common pairwise LD measures) between the two population is approximate, which indicates that the genetic backgrounds are also similar between the two pigs population. However, the mean correlation of *r* between two pigs population is only 0.538, which indicates that the LD consistency between two pigs populations was not high (Figure 1B). The detailed information can be found in a previous study (Song et al., 2019). Both in the LM and XD population, the phenotypic data consist of the total number born alive (NBA) and litter size (TNB), which were used for subsequent analysis (Table 1).

**TABLE 1:** Summary of statistics between the two populations.

Population	Trait	Number of individuals	Counts of observations	Mean	Sd	Birth year	Genotyped animals
LM	NBA	5,907	19,660	9.83	3.03	2004 to 2016	1,641
	TNB	5,907	19,660	10.85	3.06		
XD	NBA	4,842	18,369	9.88	2.94	2004 to 2015	762
	TNB	4,842	18,369	10.35	2.95		

NBA: total number born alive; TNB: litter size.

In our study, we used the corrected phenotypic values as response variables in the genomic prediction analyses. Based on the pedigree, using a single-trait repeatability model to estimate the breeding values (EBV), which was used to derived the corrected phenotypic values for these traits. The abovementioned model used to estimate EBV is documented by the Center of National Swine Genetic Evaluation of China. The model is as follows:

y=Xb+Za+Wpe+e, (1)

where y is a vector of original phenotypic values; b is the vector of the fixed effects, including herd-year-season, in which the season is divided into four levels; a is the vector of additive genetic effects;  pe is the vector of random permanent environmental effects; e is the vector of residual errors; X represents an incidence matrix relating to fixed effects; Z and W represent an incidence matrix relating to additive genetic effects and random permanent environmental effects to phenotypic records, respectively. The additive genetic effects a , random permanent environmental effects pe , and residual errors e follow the distributions as: $g\;∼\;N\left(0\;,\;\sigma_{a}^{2}A\right)$ , $pe∼N\left(0,\sigma_{pe}^{2}I\right)$ and $e\;∼\;N\left(0\;,\;\sigma_{e}^{2}I\right)$ , $\sigma_{a}^{2}$ is the additive genetic variance; $\sigma_{pe}^{2}$ is the variance of random permanent environmental effects; $\;\sigma_{e}^{2}$ is the residual variance; I is an identity matrix; and A is a relationship matrix constructed from the pedigree information. After adjustment by a single-trait repeatability model, the corrected phenotypic values (y_c_) for each trait were calculated as EBV plus the average estimated residuals over the parity of a sow. We used the DMUAI module in the DMU software (Madsen et al., 2006) for implementing the model and calculating EBV and EBV reliability.

### 2.2 Genotype Data and Imputation

A total of 2,403 sows selected from the LM (1,641) and XD 762) population were genotyped using the PorcineSNP80 BeadChip (Illumina, San Diego, CA). SNPs with a minor allele frequency (MAF) < 1%, genotyping call rate <90%, a Hardy–Weinberg equilibrium test *p*-value < 1 × 10^−7^, and the individuals whose their EBV reliability was <0.3 were removed. After quality control, the final marker dataset included 56,463 SNPs and all the genotyped individuals were retained.

In a previous work (Song et al., 2019), the 80 K chip was imputed to the WGS genotypes based on a combined reference panel using Beagle 4.1 (Browning and Browning, 2009) and the average imputation accuracy was 0.92 across all variants. The combined reference panel consists of 289 pigs from six breeds, including 46, 766, 110 SNPs as reference data for imputation (Yan et al., 2017). After genotype imputation, to control the quality of WGS data, the variants were selected on autosomes and the SNPs with a MAF <1% were removed. In addition, for all the random pairs of SNPs that were in high LD with each other (*r* ^*2*^ ≥ 0.999), we kept one of them. After the quality control, the final WGS dataset included 8,339,801 SNPs for the following analysis. The quality control was implemented using PLINK(v1.90) (Chang et al., 2015).

### 2.3 Haplotype Construction

In our study, the genotypes were phased using Beagle 4.1 before constructing the haplotypes. The method to define haplotype was based on LD between SNPs, and the haploblocks were built separately for each chromosome. The start and end points of haplotypes were designated by the way in which *r* ^*2*^ between every two SNPs in the haploblocks was greater than or equal to a threshold value, and the continuous SNPs within the point formed haplotypes. For any pairs of SNPs, the *r* ^*2*^ value was computed using PLINK(v1.90) and was derived from the following equation:

$$r^{2}=\;\frac{{\left[cov\left(g_{i},g_{j}\right)\right]}^{2}}{var\left(g_{i}\right)*var\left(g_{j}\right)},$$ (2)

where $g_{i}$ and $g_{j}$ are the genotypes which are coded as 0, 1, or 2 for SNP i and j . The *r* ^*2*^ value is standardized from zero to one, and the greater the *r* ^*2*^ value is, the higher is the LD between SNPs. The zero *r* ^*2*^ value indicates no LD and the one *r* ^*2*^ value indicates complete LD between loci. We defined a haploblock by grouping the SNPs if the LD between SNPs in this haploblock were greater than or equal to the threshold, which was set into nine levels (0.1,0.2,0.3,0.4,0.5,0.6,0.7,0.8, and 0.9). The extreme zero *r* ^*2*^ threshold indicates that a whole chromosome is selected as a haplotype block, while one indicates each individual SNP is a haplotype block.

### 2.4 Haplotype Allele Re-Code

The haplotype was constructed from the continuous SNPs throughout the region of the genome, and the haplotype alleles were treated as a pseudomarker which were recoded as 0,1, and 2 by using numerical dosage coding strategies (Meuwissen et al., 2014), which were based on the copy number of the haplotype alleles carried by the individual. The recoding of the haploblock formed by two biallelic SNPs (such as A1/A2 and B1/B2) was detailedly illustrated in Supplementary Table S1 **.** After recoding, the haplotype genotype matrix (the element is 0,1,2) was generated, in which the dimension was N×H, where N was the number of individuals and H was the total number of haplotype alleles. To investigate the influence of recoding, this study also treated a single SNP as a haplotype based on the 80 K chip data, which was then recoded as 0,1, and 2 and compared with a traditional genotype matrix based on SNP.

### 2.5 Genomic Prediction Model

Genomic prediction for NBA and TNB was performed, by constructing the relationship matrix for either SNPs or haplotypes in three models. The first model is the genomic best linear unbiased prediction (GBLUP) model, which was described by Vanraden (2008). The second model is the genomic haplotype-based best linear unbiased prediction (GHBLUP) model. The last model is based on a linear mixed model with two random genomic effects, which we termed GH + GBLUP.

#### 2.5.1 GBLUP Model

The GBLUP model was described as

y=1u+Zg+e, (3)

where y is a vector of the corrected phenotypic values; u is the overall mean; 1 is a vector of ones; g is the vector of additive genetic effects; e is the vector of residual errors; and Z represents an incidence matrix relating to the additive genetic values to phenotypic records. We assumed that the additive genetic effects g and residual errors e as random effects following the distributions as: $g\;∼\;N\left(0\;,\;\sigma_{g}^{2}G\right)$ and $e\;∼\;N\left(0\;,\;\sigma_{e}^{2}I\right)$ , $\sigma_{g}^{2}$ is the additive genetic variance; $\;\sigma_{e}^{2}$ is the residual variance; I is an identity matrix; and G is a genomic relationship matrix constructed from SNP. In our study, the genomic relationship matrix G was calculated from the following equation:

$$G=\frac{MM^{T}}{2\sum p_{i}\left(1-p_{i}\right)},$$ (4)

where M  is a matrix of the centered SNP genotypes; $M^{T}$ is a transpose matrix of M ; and $p_{i}$ is the MAF of the *i*th SNP.

#### 2.5.2 GHBLUP Model

GHBLUP is similar to GBLUP except for the genomic relationship matrix $G_{H}$ , which was constructed from the haplotypes. The genomic relationship matrix $G_{H}$ was calculated from the following equation:

$$G_{H}=\frac{M_{H}M_{H}^{T}}{2\sum p_{i}\left(1-p_{i}\right)},$$ (5)

where $M_{H}\;$ is a matrix of centered haplotype alleles; $M_{H}^{T}\;$ is a transpose matrix of M ; $p_{i}$ is the frequency of the *i*th haplotype allele.

#### 2.5.3 GH + GBLUP Model

Considering that there are higher LD between the blocked SNPs than non-blocked SNPs (single SNP that lie outside the haploblocks because of their low LD with other SNPs). Thus, the third model was based on a linear mixed model with two random genomic effects, one was captured by the haplotype alleles (constructed from blocked SNPs) and the other was by the non-blocked SNPs.

The GH + GBLUP model was described as:

$$y=1u+Zg_{block}+Zg_{non-block}+e,$$ (6)

where y, 1,  u  , and e are the same as in GBLUP; $g_{block}$ is the vector of genomic values captured by the haplotype alleles (constructed from blocked SNPs); $g_{non-block}$ is the vector of genomic values captured by non-blocked SNPs; Z is an incidence matrix that links $g_{block}$ and $g_{non-block}$ to y . We assumed that the additive genetic effects $g_{block}$ and $g_{non-block}$ as random effects following the distributions as: $\;g_{block}\;∼\;N\left(0\;,\;\sigma_{g_{block}}^{2}G_{Block}\right)$ and $g_{non-block}\;∼\;N\left(0\;,\;\sigma_{g_{non-block}}^{2}G_{Non-block}\right)$ , $\sigma_{g_{block}}^{2}$ and $\sigma_{g_{non-block}}^{2}$ are the additive genetic variance, respectively, based on the haplotype alleles and non-blocked SNPs; $G_{Block}$ and $G_{Non-block}$ are the same as in Eq. 4, Eq. 5, respectively. The variance components in GBLUP, GHBLUP, and GH + GBLUP were estimated by using the R package *regress* (Clifford and Mccullagh, 2012).

### 2.6 Evaluation of Prediction Models

The performance of genomic predictions by using different predictors (haplotype allele and individual SNP) was compared. These comparison was based on different genotype data dimensions (80 K SNP and WGS data), different population sizes (single population and multi-population), and different predictive models (GBLUP, GHBLUP, and GH + GBLUP).

To evaluate the performance of genomic prediction, the entire dataset was divided into training population and validation population according to the birth data. In this study, 223 and 196 younger animals from LM and XD were assigned to the validation population, whose birth dates were after December 2013 in LM population and after April 2013 in XD population, respectively. The remaining older animals were assigned to the training population, which was used to build prediction models. The accuracy of genomic prediction was calculated as the correlation between the predicted genomic estimated breeding value (GEBV) and the corrected phenotypic values in the validation.

## 3 Results

### 3.1 Haplotype Stastistics

**FIGURE 2:**
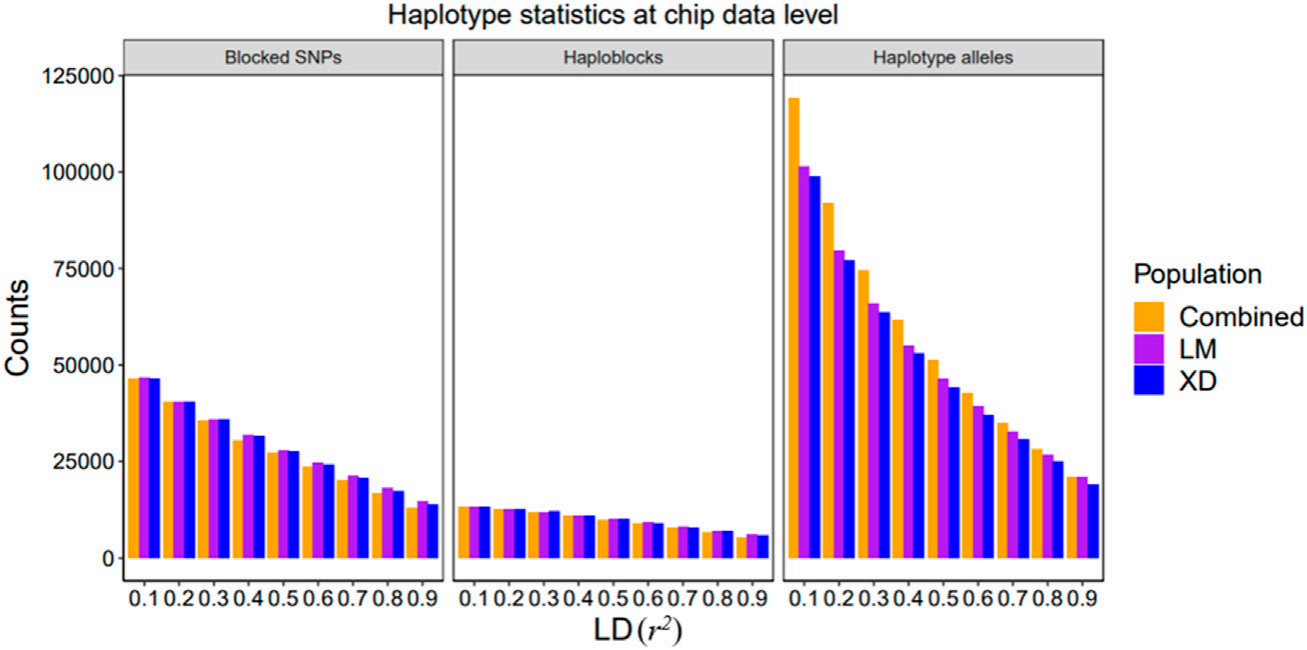
Summary of single and combined population haplotype statistics at the chip data level. The bar plot of the counts of blocked SNPs, haploblocks, and haplotype alleles in different LD threshold value, respectively.

The details about the statistics of haplotypes constructed based on 80 K SNP and WGS data in the single-population and multi-population are presented in Figure 2 and Supplementary Figure S1. The haplotype statistics include the haplotype alleles (variables), haploblocks, and blocked SNPs at different *r* ^*2*^ value levels. With the increase of *r* ^*2*^, the number of haplotype alleles, haploblocks, and blocked SNPs decrease, indicating that the higher the LD level, the more difficult to build haplotypes, and when the threshold approaches 1, a haplotype is approximately composed of an individual SNP. In addition, there are no significant differences in haploblocks and blocked SNPs between the combined and single population, even fewer in the combined population, but there are importantly more haplotype alleles in combined population than in single populations, suggesting that more information can be gained from the haplotype constructed in the combined population. In our results, the haplotype statistics based on both 80 K chip data and sequence data follow the abovementioned rules.

### 3.2 Genomic Prediction Accuracy

**FIGURE 3:**
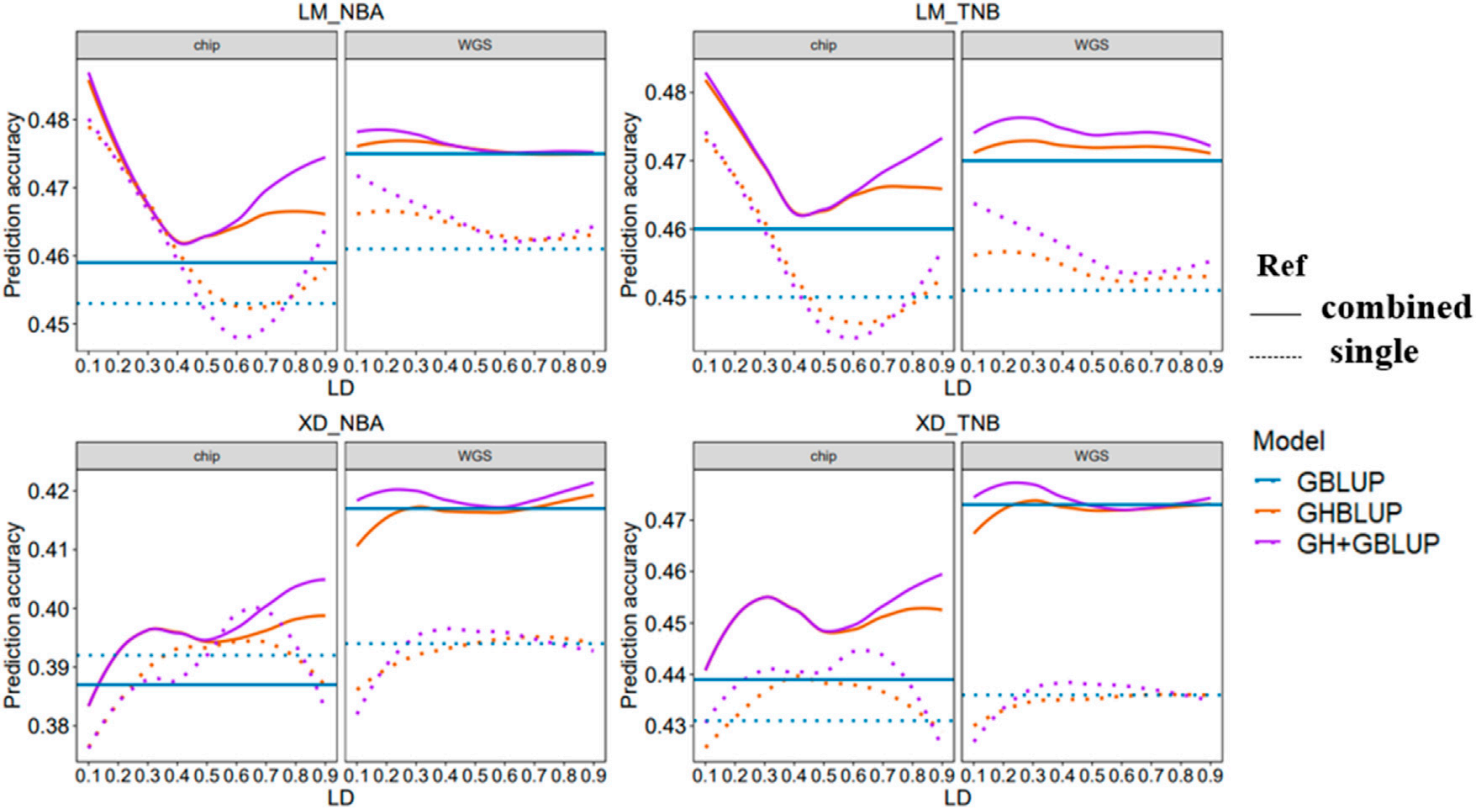
Genomic prediction accuracy of all scenarios with different *r* ^*2*^ thresholds. The left side shows the trend of accuracy predicted using the 80 K chip data while the right side using the WGS data. The different colored lines represent different prediction methods in which blue, orange, and purple line represent GBLUP, GHBLUP, and GH + GBLUP, respectively. The solid line represents the prediction accuracy trend of combined population, while the dotted line represents the single population.

In this study, we evaluated the accuracy of GS of two reproduction traits in eight different scenarios which differed in the maker density (80 K chip or WGS), the reference population sizes (single population or combined population), and explanatory variables (SNP or haplotype) ((Figure 3)).

#### 3.2.1 Comparison of Accuracies of GS Between Chip Data and WGS Data

Our result showed that the accuracies of genomic predictions within-population using the WGS data were inferior to 80 K chip data in most scenarios. When the maker density was increased from the 80 K chip data to the WGS data, for LM population, the accuracy of GBLUP had a small increase for NBA (0.453–0.461) and no change for TNB, while the accuracy of GHBLUP and GH + GBLUP had worse performance on *r* ^*2*^ < 0.3 for both trait. For the XD population, there were no obvious differences in the accuracy of GBLUP, while the accuracy of GHBLUP and GH + GBLUP had small decrease both for NBA and TNB.

#### 3.2.2 Comparison of Accuracies of GS Between Individual SNP and Haplotype Alleles

When using the within-population as the reference population, the accuracy of genomic prediction using the haplotype alleles was increased compared to the individuals SNP whether using 80 K chip data or WGS data.

For using 80 K chip data to predict LM population, the accuracy of GHBLUP and GH + GBLUP had significant improvement compared to GBLUP. The maximum increase is 5.7 and 6.0% for NBA and 5.1 and 5.3% for TNB. For predicting XD population using 80 K chip data, the accuracy of GHBLUP and GH + GBLUP had a slight increase compared to GBLUP. The maximum increase is only 0.8 and 2.3% for NBA and 1.9 and 3.2% for TNB.

When genomic prediction using the WGS data, for predicting LM population within-population, the accuracy of GHBLUP and GH + GBLUP had respectively an increase of 1.3 and 2.4% for NBA and of 1.3 and 2.9% for TNB compared to GBLUP. While for predicting XD population within-population, the accuracy of GHBLUP and GH + GBLUP had no change for NBA and TNB.

#### 3.2.3 Comparison of Accuracies of GS Between Single Population and Combined Population

When the reference population was enlarged from the single population to combined population for genomic prediction using the 80 K chip data, the accuracy of GBLUP in LM population slightly increased from 0.453 to 0.450 to 0.459 and 0.460 for NBA and TNB, respectively. For the XD population, the accuracy of GBLUP decreased for NBA and increased for TNB. Correspondingly, when using the WGS data, the accuracy of GBLUP had importantly improved from 0.461 and 0.451 to 0.475 and 0.470 for LM population and from 0.394 and 0.436 to 0.417 and 0.473 for XD population.

When genomic prediction used the haplotype alleles based on 80 K chip data, for LM population, the accuracy of GHBLUP and GH + GBLUP in combined population both had improved at all *r* ^*2*^ thresholds compared to single population, while the maximum accuracy of GHBLUP and GH + GBLUP both increased 1.3% for NBA and 1.7% for TNB. For the XD population, the accuracy of GHBLUP and GH + GBLUP in combined population had improved at most of the *r* ^*2*^ thresholds for NBA, and at all *r* ^*2*^ thresholds for TNB compared to single population while the maximum accuracy of GHBLUP and GH + GBLUP increased 1.3 and 1.2% for NBA and 4.3 and 3.1% for TNB, respectively. Correspondingly, based on the WGS data, when the reference population was enlarged from single population to combined population, for the LM population, the maximum accuracy of GHBLUP and GH + GBLUP increased to 2.1 and 1.5% for NBA and 3.5 and 2.8% for TNB. For the XD population, the maximum accuracy of GHBLUP and GH + GBLUP increased to 6.1 and 6.3% for NBA and 8.7 and 9.1% for TNB, respectively.

When genomic prediction using the 80 K chip data in combined population is carried out, compared to GBLUP, the accuracy of GHBLUP and GH + GBLUP had important improvement at all *r* ^*2*^ thresholds for NBA and TNB for the LM population, while the maximum accuracy of GHBLUP and GH + GBLUP increased to 5.7 and 5.9% for NBA and 4.6 and 4.9% for TNB, respectively. Correspondingly, when used the WGS data, GH + GBLUP had the highest accuracy on the three models and the accuracy of GHBLUP had slightly increased compared to GBLUP, and the maximum accuracy of GHBLUP and GH + GBLUP increased to 0.4 and 0.8% for NBA and 0.6 and 1.5% for TNB, respectively. For the XD population, we found a similar trend. When used the 80 K chip data, compared to GBLUP, the maximum accuracy of GHBLUP and GH + GBLUP increased to 3.4 and 4.9% for NBA and 4.3 and 4.6% for TNB, respectively. Correspondingly, when used WGS data, the maximum accuracy of GHBLUP and GH + GBLUP increased to 0.5 and 1.0% for NBA and 0.2 and 1.1% for TNB, respectively.

According to our results, GH + GBLUP had the best performance on genomic prediction and using GH + GBLUP based on the WGS data in multi-population displayed better genomic prediction accuracy for most scenarios. In this scenarios, we found that the building haploblock based on low LD threshold (*r* ^*2*^ = 0.2–0.3) had the highest genomic prediction accuracy among the different LD thresholds for reproduction traits in Yorkshire pig population.

### 3.3 Regression Coefficient of Genomic Prediction

**FIGURE 4:**
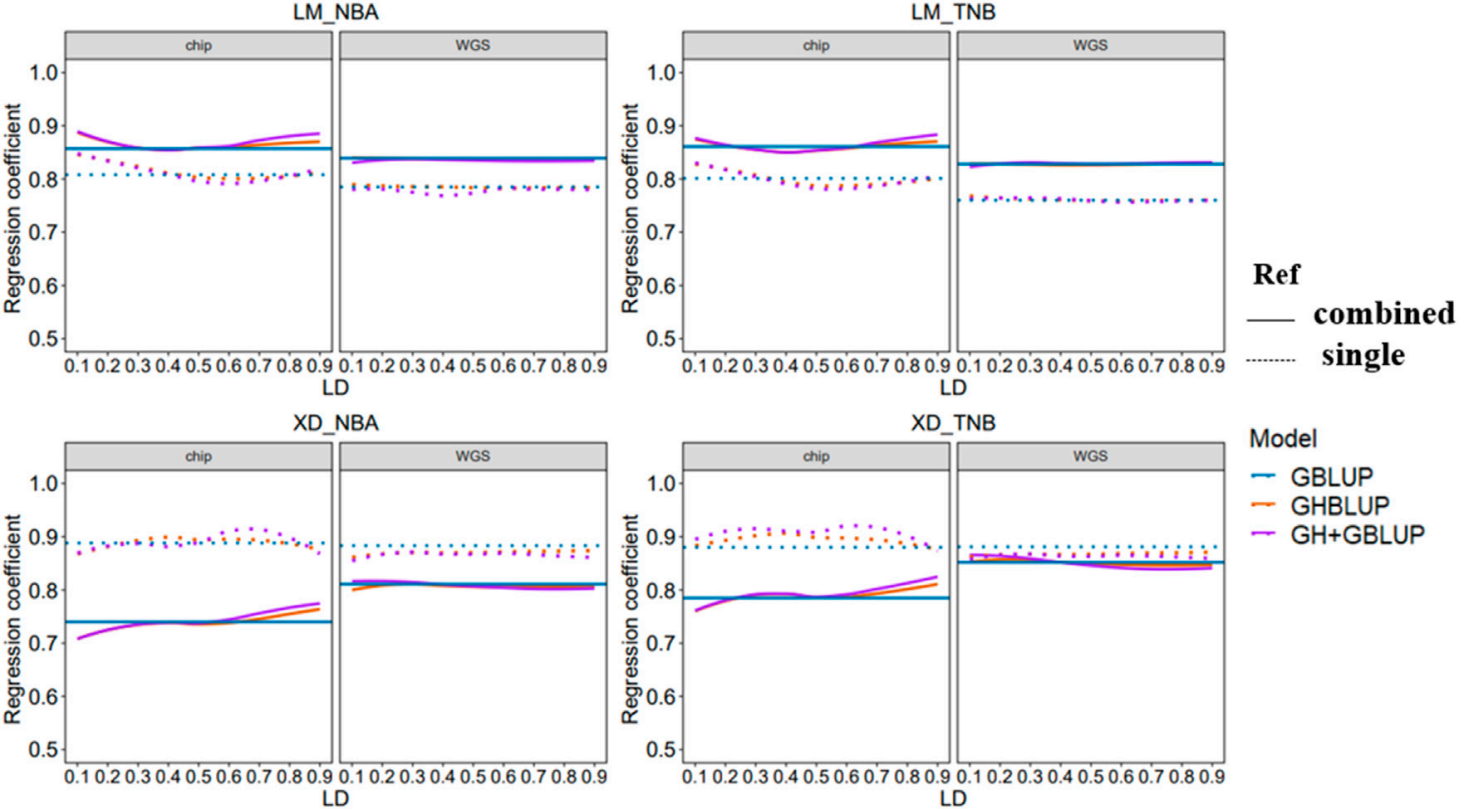
Regression coefficient of all scenarios with different *r* ^*2*^ thresholds. The left side shows the trend of accuracy predicted using the 80 K chip data while the right side using the WGS data. The different colored lines represent different prediction methods in which blue, orange, and purple line represent GBLUP, GHBLUP, and GH + GBLUP, respectively. The solid line represents the prediction accuracy trend of combined population, while the dotted line represents the single population.

The regression coefficient of genomic prediction was assessed using the slope of the regression of the adjusted phenotype on the GEBV. In our study, the regression coefficients were presented in Figure 4. When the reference population was the combined population for predicting the LM population, the regression coefficients were closer to 1 compared with within-population. However, the trend of regression coefficient was reversed in predicting the XD population. In addition, regression coefficients using the WGS data based on three methods almost had no change for different LD levels whether predicted in within-population or multi-population.

### 3.4 Influence of Haplotype Allele Re-Code

In our study, when a single SNP was treated as one haplotype (GHBLUP_SNP1), and compared with classical GBLUP, our results showed that the accuracy and regression coefficient of GHBLUP were equal to GBLUP (Table 2). In addition, the genetic variance and residual variance of GHBLUP were also consistent with GBLUP.

**TABLE 2:** The comparison of prediction performance of the two methods.

Val	Ref	Trait	Method	Acc	Regression coefficient	Genetic variance	Residual variance
LM	LM	NBA	GBLUP	0.453	0.808	1.643	0.569
LM	LM	NBA	GHBLUP_SNP1	0.453	0.807	1.642	0.569
LM	Combined	NBA	GBLUP	0.459	0.857	1.193	0.491
LM	Combined	NBA	GHBLUP_SNP1	0.458	0.857	1.193	0.491
LM	LM	TNB	GBLUP	0.450	0.801	2.285	0.751
LM	LM	TNB	GHBLUP_SNP1	0.450	0.801	2.284	0.751
LM	Combined	TNB	GBLUP	0.460	0.861	1.634	0.624
LM	Combined	TNB	GHBLUP_SNP1	0.460	0.861	1.634	0.624
XD	XD	NBA	GBLUP	0.392	0.888	0.601	0.260
XD	XD	NBA	GHBLUP_SNP1	0.392	0.888	0.601	0.260
XD	Combined	NBA	GBLUP	0.387	0.740	1.193	0.504
XD	Combined	NBA	GHBLUP_SNP1	0.387	0.740	1.193	0.504
XD	XD	TNB	GBLUP	0.431	0.880	0.806	0.259
XD	XD	TNB	GHBLUP_SNP1	0.431	0.880	0.806	0.259
XD	Combined	TNB	GBLUP	0.439	0.785	1.636	0.653
XD	Combined	TNB	GHBLUP_SNP1	0.440	0.785	1.635	0.653

Val: validation set of population; Ref: reference set of population; Acc: the prediction accuracy; NBA: total number born alive; TNB: litter size; GHBLUP_SNP1: the method of treating a single SNP, as a haplotype.

## 4 Discussion

For the multi-population, the previous studies had found that using the individual SNP method based on WGS data (Iheshiulor et al., 2016; Ye et al., 2020) or haplotype method based on chip data (Cuyabano et al., 2015b; Hess et al., 2017), which enhances the ability to capture LD between the variant and QTLs, can effectively improve the accuracy of genomic prediction. In our study, we evaluated the impact of the WGS data, haplotype method, and combined population on GS. For within-population GS, our results presented, using the WGS data, were inferior to 80 K chip data in most scenarios, while using the haplotype method can improve the accuracy compared to SNP whether using the 80 K chip data or WGS data. For multi-population GS, our results were consistent with previous reports that using the WGS data or constructing haplotype can improve the prediction accuracy. In addition, we found that simultaneously combining haplotype method and WGS data could yield better performance for the multi-population genomic prediction.

### 4.1 Genomic Prediction Performance of Different Marker Densities

In this study, we compared the accuracy of genomic prediction based on the 80 K chip SNP data vs the WGS data to evaluate the GS performance of different marker densities. According to previous studies, it is theoretical that the accuracy of genomic prediction is expected to improve by using the WGS data compared with using the chip SNP data (Meuwissen and Goddard, 2010; Druet et al., 2014; Hayes et al., 2014), because the WGS data contains higher marker density, more causal mutations, which results in a high level of LD between SNPs and QTL. In the simulation data, the prediction accuracy increased within a population based on the WGS data (Macleod et al., 2014; Yan et al., 2017). However, these predicted results using the real data have not been observed in practice, for example, a recent study found that the accuracy of genomic prediction was not increased when using the imputed sequence data in Holstein Friesian cattle (van Binsbergen et al., 2015). Our result presented that the WGS data had better performance than the chip data when using the GBLUP method for the within-population genomic prediction. The similar result was reported in Brown Swiss Cattle for the trait of nonreturn rate in heifers (Frischknecht et al., 2018).

For the GHBLUP and GH + GBLUP model, our result also presented that GS within a population based on the WGS data had decreased the accuracy compared with the chip data. This is consistent with the result reported in Chinese Simmental beef cattle (Li et al., 2021). It is possible that increasing SNP density can produce the number of identified haplotype alleles, which includes some rare haplotype alleles, and thus shrink the effect of these alleles toward zero when calculating the genetic effect (Gianola, 2013). Hence, the haplotype approach may not improve the prediction accuracy within a population when marker density increased from the chip data to the WGS data.

A previous study has shown that the higher the imputation accuracy, the higher is the prediction accuracy (Nasir et al., 2015). The imputation accuracy is influenced by several factors including marker density, imputation algorithms, reference population size, and the structure of the target population (Hayes et al., 2012; Ye et al., 2018). Thus, we consider that the abovementioned factors to impute genotype is an attractive strategy for genomic prediction. In addition, whether the better predictive performance based on the data after imputation depends on several factors such as LD, MAF, and genotyping errors (Iwata and Jannink, 2010; Zhang and Druet, 2010; Ye et al., 2019).

### 4.2 Potential Impact of the Haplotype Method on Genomic Prediction

In our study, we compared the genomic prediction performance of three models (GBLUP, GHBLUP, and GH + GBLUP). To date, the genomic prediction by constructing haplotype based on the WGS data in pigs has rarely been investigated.

For the prediction based on the chip data within-population, our results showed that GH + GBLUP method had the best performance of prediction, followed by GHBLUP, which indicated that the explanatory variables based on haplotypes had certain advantages compared to the individual SNP. Some studies have reported similar findings while using haplotypes in genomic predictions. In the study of Hess et al. (2017), their results showed that the prediction accuracy increased when used the fixed-length haplotype than single SNP in admixed New Zealand dairy cattle population. Cuyabano et al. (2014, 2015a) used LD information to construct haplotype and reported that haplotype method based on the average LD threshold (*r* ^*2*^ ≥ 0.45) can increase the prediction accuracy for milk production traits in the Nordic Holstein population. Similarly, Teissier et al. (2020) reported that using LD-haplotype also had a better prediction performance in French dairy goats. The advantage of haplotype method can be explained by the assumption that haplotypes are in stronger LD with the causative mutation than the individual SNP, because a QTL is in complete LD with a multimarker haplotype while not in complete LD with any individual SNP. The haploblocks consist of multiple loci, when a mutation occurred in a loci of a haploblock, SNP allele frequencies had changed little while haplotype allele frequencies had changed more, so the haplotypes can better capture mutations compared to single loci (Curtis et al., 2012). In addition, the fitting explanatory variables for haplotype alleles instead of individual SNP can improve the ability to capture short-range epistatic effects between the loci within the same haploblock (Yong et al., 2018).

As for the WGS data within-population, our result shows that fitting covariates for haplotypes rather than SNPs could increase the prediction accuracy but the increase is slight compared to the chip data. The increase becoming smaller can be explained as mentioned previously that the increased SNP density can produce the number of identified haplotype alleles, which include some rare haplotype alleles, and thus shrink the effect of these alleles toward zero when calculating the genetic effect (Li et al., 2021). Another reason could be explained that when the marker density is high enough, the physical location range of a haploblock constructed based on LD may approximate a single marker locus, resulting in the LD between haplotype and QTL may be close to the LD between a single marker and QTL. In addition, the capture of mutations and short-range epistatic may be ineffective because too many SNPs with high LD may be considered as noise (Song et al., 2019). Thus, the advantage of the haplotype method compared to the individual SNP may become weak for the WGS data. One possible way to solve this problem is to reasonably reduce the dimension of the WGS data. Previous studies suggested that preselected potential causal markers or QTL obtained from the WGS data can improve the accuracy of genomic prediction (Raymond et al., 2018; Song et al., 2019; Ye et al., 2020). Although these studies are based on the individual SNP, the prediction based on haplotype using this strategy is expected to improve the accuracy. Gao et al. (2017) had reported that incorporating gene annotation into the haplotype-based method according to gene positions, which reduce the density of WGS data, had better performance in genomic prediction in the *Drosophila* Genetic Reference Panel. Thus, the evaluation of the impact of constructing haplotype based on the preselected WGS data is worth to be further explored in livestock.

In our study, the result shows that the GH + GBLUP method had the best performance on genomic prediction based on both the WGS data and chip data. Considering that there are higher LD between blocked SNPs than non-blocked SNPs, so the GH + GBLUP was based on a linear mixed model with two random genomic effects which is similar to the Kernel Averaging model (Gustavo et al., 2010), giving weight to each random genomic effects according to their capture of genetic variation. Therefore, the possible reason why the prediction of accuracy of GH + GBLUP is higher than GHBLUP is that GH + GBLUP can give an appropriate weight to the blocked SNPs and non-blocked SNPs.

### 4.3 Combined Population Genomic Prediction

The size of the reference population and the relationship between the reference and validation populations are two key factors that can improve the multi-population genomic selection, which had been reported in some previous studies (Brendum et al., 2011; Lund et al., 2011).

Our result shows that the multi-population genomic prediction, using GBLUP method based on the 80 K chip data, achieves a higher accuracy compared with the within-population prediction, except for predicting NBA in XD population. The reason why the phenomenon occurs in that XD population with NBA can explain that the phase difference is large enough between a tagging SNP and a large QTL in the two population for the target trait (Saatchi et al., 2014), and the GBLUP method lack power to capture the population-specific effects. This is one of the factors that limit the improvement in prediction accuracy for some traits when predicting in multi-population.

For the haplotype method based on the 80 K chip data, our result shows that the accuracy of multi-population genomic prediction had improved compared with the within-population, including predicting NBA in XD population as opposed to using the GBLUP method. This may be explained by the following fact: when the constructed haplotype is in multi-population, the population-specific haplotype alleles are generated, which are present in one population and not in another. Fitting covariates for haplotype have a better ability to capture the population-specific effects than SNPs if the population-specific haplotype alleles contain population-specific QTL. Hess et al. (2017) reported that combined admixed reference population can increase the prediction accuracy when using the fixed-length haplotype method compared to the within-population prediction. Cuyabano et al. (2015b) also reported a similar result but using the variable-length haplotype. This indicated that using the haplotype method in multi-population prediction may potentially improve the accuracy.

In our study, we evaluated the haplotype method for multi-population genomic prediction based on the WGS data. For multi-population genomic prediction using the haplotype method, our result shows that prediction based on the WGS data has better performance compared to the chip data, which is contrary to the result of single-population using the haplotype method. It is possible that as the size of the reference population increases, the information of phenotypic data becomes sufficient to detect causative mutations by the haplotype alleles, which reduces the number of rare haplotype whose effects are shrunk toward zero, while increasing the number of effective haplotype alleles. This allows us to accurately estimate the genomic breeding value of animal, and improve the prediction accuracy. In addition, incorporating causative mutations into the haplotypes will enhance the ability to detect similar QTL which segregates between population. Compared with the SNP panels, the WGS data will improve the ability to differentiate the sequence-resolution haplotype alleles within a haploblock (Hess et al., 2017), while all the true haplotype alleles including causative mutations in the dataset can theoretically be identified between multi-population at the sequence level. It would better assess what extend genetic variance due to haplotype effects is specific within-population or common among populations. In addition, we have found that using the haplotype method based on the WGS data in combined population has better GS performance in most scenarios, and in this scenario that building haploblock based on low LD threshold (*r* ^*2*^ = 0.2–0.3) produced an optimal set of variables for reproduction traits in Yorkshire pig population. Similar as previous study (Cuyabano et al., 2014), our result also revealed that to achieve better prediction accuracy, the optimum LD threshold could be considered when using the haplotype method for reproduction traits in Yorkshire pig population, which can be used as reference for genomic prediction considering LD.

### 4.4 Impact of Haplotype Allele Re-Code by Using Numerical Dosage Coding Strategies of GS

In our study, to investigate the impact of haplotype alleles recode, we compared the performance of GHBLUP_SNP1 and GBLUP based on the 80 K chip data. There was no difference between the performance of GHBLUP_SNP1 and GBLUP, which was what we expected. A single SNP locus is a biallelic locus, the information it carries is determined by the frequency of two alleles on its biallelic loci. The frequency of haplotype alleles is equal to the frequency of alleles on the biallelic loci, while treating a single SNP as a haplotype and was recoded by using numerical dosage coding strategies, which are based on the copy number of haplotype alleles carried by the individual. In other words, the SNP locus information is determined by the frequency of alleles, while the haplotype information is determined by the copy number of the haplotype alleles, which is divided by population size and is equal to the frequency of alleles on biallelic loci. Therefore, recoding by using numerical dosage coding strategies did not increase or decrease the information of loci, which has no impact on GS.

### 4.5 Variable-Length Versus Fixed-Length Haplotype

Our study evaluated haplotypes termed as variable-length haplotype that were based on different LD levels thresholds in two Yorkshire pig population. The methods to define haploblock that group together consecutive SNPs or a fixed range of SNPs across the genome, termed fixed-length haplotype. The variable-length haplotype involving the calculation of LD usually reflects the characteristics of the genome better than the artificially defined fixed-length haplotype. A previous study has reported different recombination across the genome in many species (Nachman, 2002), indicating that the variable-haplotype is more likely inherited together, which suggests that the haploblock length for genomic prediction may differ across the genome. Another reason why the optimal haploblock lengths may differ across the genome is the artificial selection which has resulted in some regions around QTL undergoing selective sweeps (Hess et al., 2017). Therefore, the variable-length haplotype theoretically has better performance on genomic prediction than the fixed-length haplotype, while the variable-length haplotype is more complicated and time-consuming. In addition, the optimal size defining haplotype for genomic prediction depends on the distance between the SNPs and the LD structure of the population (Calus et al., 2008). Thus, the optimal fixed-length or LD threshold for genomic prediction needs to be evaluated for each dataset independently.

## 5 Conclusion

Our study suggested that using the haplotype method based on the chip data can effectively increase the prediction accuracy of both within-population and multi-population compared to the individual SNP method, especially had better prediction performance in multi-population. Comparing to the chip data, using the individual SNP method based on the WGS data can improve the accuracy of prediction in multi-population but not within-population. In addition, we found that simultaneously combining the haplotype method and WGS data could yield better performance for multi-population genomic prediction, and in this scenario that it was optimal to determine low LD threshold to build haploblocks for the reproduction traits in Yorkshire pig population.

## Data Availability

The original contributions presented in the study are included in the article/Supplementary Material. Further inquiries can be directed to the corresponding author.
