# Supplementary material for: Genomic Prediction Using LD-Based Haplotypes in Combined Pig Populations
Source: Front Genet. 2022 Jun 9;13:843300. doi: 10.3389/fgene.2022.843300 (PMC9218795; doi:10.3389/fgene.2022.843300)
Supplement: Supplementary file 1 [file DataSheet1.docx]

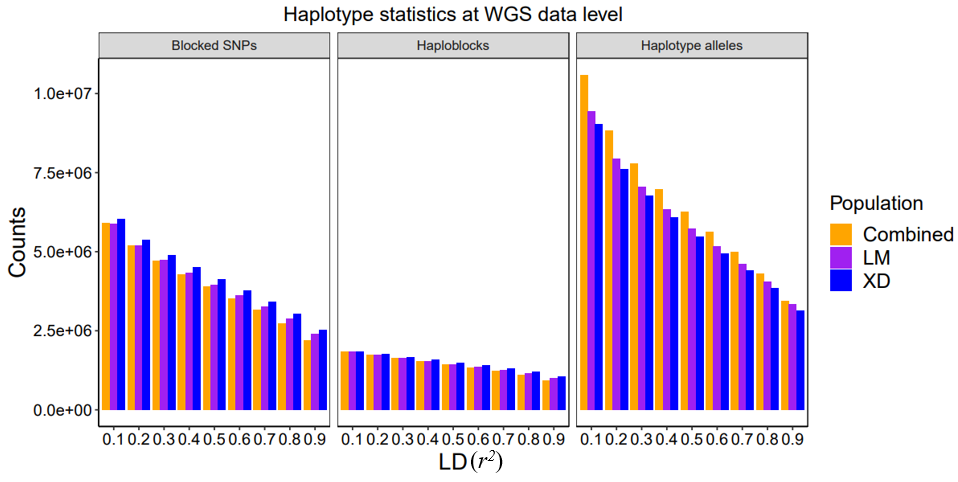


**Figure S1.** Summary of single and combined population Haplotype statistics at WGS data level. The bar plot of the counts of blocked SNPs, haploblocks and haplotype alleles in different LD threshold value, respectively.

**TABLE S1.** The re-coded of a haploblock formed by two bi-allelic SNPs

| Individual | Haplotype  Allele 1 | Haplotype  Allele 2 | Re-coded of all haplotype alleles of the haploblock | | | |
| --- | --- | --- | --- | --- | --- | --- |
|  | | | A1B1 | A1B2 | A2B1 | A2B2 |
| N1 | A1B1 | A1B2 | 1 | 1 | 0 | 0 |
| N2 | A1B1 | A2B1 | 1 | 0 | 1 | 0 |
| N3 | A1B1 | A2B2 | 1 | 0 | 0 | 1 |
| N4 | A1B2 | A2B1 | 0 | 1 | 1 | 0 |
| N5 | A1B2 | A2B2 | 0 | 1 | 0 | 1 |
| N6 | A2B1 | A2B2 | 0 | 0 | 1 | 1 |
| N7 | A1B1 | A1B1 | 2 | 0 | 0 | 0 |
| N8 | A1B2 | A1B2 | 0 | 2 | 0 | 0 |
| N9 | A2B1 | A2B1 | 0 | 0 | 2 | 0 |
| N10 | A2B2 | A2B2 | 0 | 0 | 0 | 2 |

A1B1, A1B2, A2B1, A2B2 are four haplotype alleles of the haplotype; Ni is ith individual.

**TABLE S2.** Predictive accuracies for all scenarios.

| **Density** | | **Model** | ${\boldsymbol{r}^{\boldsymbol{2}}}^{1}$ | | **Val_LM** | | | | **Val_XD** | | | |
| --- | --- | --- | --- | --- | --- | --- | --- | --- | --- | --- | --- | --- |
|  |  |  |  |  | **Ref_LM** | | **Ref_Combined** | | **Ref_XD** | | **Ref_Combined** | |
|  |  |  |  |  | **NBA** | **TNB** | **NBA** | **TNB** | **NBA** | **TNB** | **NBA** | **TNB** |
| 80k | GBLUP | | | - | 0.453 | 0.450 | 0.459 | 0.460 | 0.392 | 0.431 | 0.387 | 0.439 |
|  | GHBLUP | | | 0.1 | 0.479 | 0.473 | 0.485 | 0.481 | 0.377 | 0.427 | 0.383 | 0.441 |
|  |  |  |  | 0.2 | 0.474 | 0.468 | 0.478 | 0.478 | 0.382 | 0.428 | 0.393 | 0.45 |
|  |  |  |  | 0.3 | 0.469 | 0.462 | 0.465 | 0.468 | 0.391 | 0.439 | 0.399 | 0.458 |
|  |  |  |  | 0.4 | 0.460 | 0.452 | 0.464 | 0.463 | 0.393 | 0.439 | 0.392 | 0.45 |
|  |  |  |  | 0.5 | 0.455 | 0.448 | 0.461 | 0.461 | 0.393 | 0.438 | 0.398 | 0.45 |
|  |  |  |  | 0.6 | 0.453 | 0.446 | 0.466 | 0.467 | 0.394 | 0.438 | 0.392 | 0.447 |
|  |  |  |  | 0.7 | 0.452 | 0.447 | 0.465 | 0.465 | 0.395 | 0.437 | 0.397 | 0.452 |
|  |  |  |  | 0.8 | 0.455 | 0.449 | 0.467 | 0.466 | 0.391 | 0.433 | 0.4 | 0.454 |
|  |  |  |  | 0.9 | 0.458 | 0.453 | 0.466 | 0.466 | 0.387 | 0.429 | 0.398 | 0.452 |
|  | GH+GBLUP | | | 0.1 | 0.480 | 0.474 | 0.486 | 0.482 | 0.376 | 0.431 | 0.383 | 0.441 |
|  |  |  |  | 0.2 | 0.474 | 0.468 | 0.479 | 0.479 | 0.384 | 0.436 | 0.393 | 0.45 |
|  |  |  |  | 0.3 | 0.467 | 0.460 | 0.465 | 0.468 | 0.39 | 0.444 | 0.399 | 0.458 |
|  |  |  |  | 0.4 | 0.459 | 0.451 | 0.464 | 0.463 | 0.386 | 0.438 | 0.392 | 0.45 |
|  |  |  |  | 0.5 | 0.453 | 0.446 | 0.461 | 0.461 | 0.391 | 0.441 | 0.398 | 0.45 |
|  |  |  |  | 0.6 | 0.446 | 0.443 | 0.467 | 0.468 | 0.401 | 0.444 | 0.394 | 0.448 |
|  |  |  |  | 0.7 | 0.451 | 0.447 | 0.468 | 0.466 | 0.399 | 0.445 | 0.401 | 0.454 |
|  |  |  |  | 0.8 | 0.455 | 0.450 | 0.474 | 0.472 | 0.393 | 0.436 | 0.406 | 0.458 |
|  |  |  |  | 0.9 | 0.464 | 0.457 | 0.474 | 0.473 | 0.383 | 0.426 | 0.404 | 0.459 |
| WGS | GBLUP | | | - | 0.461 | 0.451 | 0.475 | 0.470 | 0.394 | 0.436 | 0.417 | 0.473 |
|  | GHBLUP | | | 0.1 | 0.466 | 0.456 | 0.476 | 0.471 | 0.386 | 0.430 | 0.410 | 0.467 |
|  |  |  |  | 0.2 | 0.467 | 0.457 | 0.477 | 0.473 | 0.390 | 0.433 | 0.417 | 0.473 |
|  |  |  |  | 0.3 | 0.466 | 0.456 | 0.477 | 0.473 | 0.392 | 0.435 | 0.417 | 0.474 |
|  |  |  |  | 0.4 | 0.465 | 0.455 | 0.476 | 0.472 | 0.393 | 0.435 | 0.416 | 0.472 |
|  |  |  |  | 0.5 | 0.464 | 0.453 | 0.476 | 0.472 | 0.394 | 0.435 | 0.417 | 0.472 |
|  |  |  |  | 0.6 | 0.463 | 0.452 | 0.475 | 0.472 | 0.395 | 0.436 | 0.416 | 0.472 |
|  |  |  |  | 0.7 | 0.462 | 0.453 | 0.475 | 0.472 | 0.395 | 0.436 | 0.417 | 0.472 |
|  |  |  |  | 0.8 | 0.463 | 0.453 | 0.475 | 0.472 | 0.395 | 0.436 | 0.419 | 0.473 |
|  |  |  |  | 0.9 | 0.463 | 0.453 | 0.475 | 0.471 | 0.394 | 0.436 | 0.419 | 0.473 |
|  | GH+GBLUP | | | 0.1 | 0.472 | 0.464 | 0.478 | 0.474 | 0.382 | 0.427 | 0.418 | 0.474 |
|  |  |  |  | 0.2 | 0.469 | 0.461 | 0.479 | 0.476 | 0.390 | 0.433 | 0.421 | 0.478 |
|  |  |  |  | 0.3 | 0.468 | 0.460 | 0.478 | 0.477 | 0.396 | 0.438 | 0.420 | 0.477 |
|  |  |  |  | 0.4 | 0.466 | 0.458 | 0.476 | 0.474 | 0.396 | 0.438 | 0.418 | 0.474 |
|  |  |  |  | 0.5 | 0.464 | 0.455 | 0.476 | 0.474 | 0.396 | 0.438 | 0.418 | 0.473 |
|  |  |  |  | 0.6 | 0.462 | 0.454 | 0.475 | 0.474 | 0.396 | 0.438 | 0.417 | 0.472 |
|  |  |  |  | 0.7 | 0.462 | 0.453 | 0.475 | 0.474 | 0.395 | 0.437 | 0.418 | 0.472 |
|  |  |  |  | 0.8 | 0.464 | 0.455 | 0.476 | 0.474 | 0.393 | 0.436 | 0.421 | 0.474 |
|  |  |  |  | 0.9 | 0.464 | 0.455 | 0.475 | 0.472 | 0.393 | 0.435 | 0.421 | 0.474 |

Val_LM = validation set from the LM Yorkshire population; Val_XD = validation set from the XD Yorkshire population; Ref_LM = only using the LM Yorkshire population as reference population; Ref_ALL = using the LM and XD Yorkshire population as reference population; Ref_XD = only using the XD Yorkshire population as reference population; NBA = number of piglets born alive; TNB = total number of piglets born; 80K = 80K SNP panel; WGS = imputed whole-genome sequencing data;

^1^The nine different LD thresholds set from $r^{2}\geq0.1$ to $r^{2}\geq0.9$.

**TABLE S****3.** Regression coefficients of pre-adjusted phenotypes on GEBVs for all scenarios.

| **Density** | | **Model** | ${\boldsymbol{r}^{\boldsymbol{2}}}^{1}$ | | **Val_LM** | | | | **Val_XD** | | | |
| --- | --- | --- | --- | --- | --- | --- | --- | --- | --- | --- | --- | --- |
|  |  |  |  |  | **Ref_LM** | | **Ref_Combined** | | **Ref_XD** | | **Ref_Combined** | |
|  |  |  |  |  | **NBA** | **TNB** | **NBA** | **TNB** | **NBA** | **TNB** | **NBA** | **TNB** |
| 80k | GBLUP | | | - | 0.808 | 0.801 | 0.857 | 0.861 | 0.888 | 0.880 | 0.740 | 0.785 |
|  | GHBLUP | | | 0.1 | 0.846 | 0.828 | 0.885 | 0.873 | 0.871 | 0.887 | 0.709 | 0.762 |
|  |  |  |  | 0.2 | 0.834 | 0.818 | 0.877 | 0.869 | 0.872 | 0.881 | 0.722 | 0.774 |
|  |  |  |  | 0.3 | 0.825 | 0.809 | 0.850 | 0.849 | 0.899 | 0.910 | 0.740 | 0.796 |
|  |  |  |  | 0.4 | 0.810 | 0.793 | 0.860 | 0.855 | 0.898 | 0.904 | 0.733 | 0.790 |
|  |  |  |  | 0.5 | 0.802 | 0.786 | 0.856 | 0.850 | 0.893 | 0.898 | 0.741 | 0.787 |
|  |  |  |  | 0.6 | 0.802 | 0.789 | 0.860 | 0.859 | 0.894 | 0.896 | 0.733 | 0.784 |
|  |  |  |  | 0.7 | 0.800 | 0.789 | 0.864 | 0.864 | 0.897 | 0.897 | 0.748 | 0.796 |
|  |  |  |  | 0.8 | 0.806 | 0.794 | 0.868 | 0.866 | 0.883 | 0.884 | 0.757 | 0.801 |
|  |  |  |  | 0.9 | 0.812 | 0.801 | 0.870 | 0.871 | 0.877 | 0.877 | 0.763 | 0.811 |
|  | GH+GBLUP | | | 0.1 | 0.848 | 0.83 | 0.887 | 0.875 | 0.870 | 0.896 | 0.709 | 0.763 |
|  |  |  |  | 0.2 | 0.833 | 0.818 | 0.878 | 0.870 | 0.879 | 0.905 | 0.722 | 0.775 |
|  |  |  |  | 0.3 | 0.822 | 0.805 | 0.850 | 0.849 | 0.895 | 0.926 | 0.740 | 0.797 |
|  |  |  |  | 0.4 | 0.807 | 0.79 | 0.860 | 0.855 | 0.877 | 0.901 | 0.735 | 0.791 |
|  |  |  |  | 0.5 | 0.798 | 0.782 | 0.856 | 0.850 | 0.884 | 0.912 | 0.741 | 0.787 |
|  |  |  |  | 0.6 | 0.787 | 0.78 | 0.863 | 0.861 | 0.917 | 0.917 | 0.740 | 0.790 |
|  |  |  |  | 0.7 | 0.799 | 0.789 | 0.871 | 0.867 | 0.913 | 0.923 | 0.759 | 0.804 |
|  |  |  |  | 0.8 | 0.806 | 0.794 | 0.884 | 0.878 | 0.893 | 0.896 | 0.768 | 0.812 |
|  |  |  |  | 0.9 | 0.819 | 0.806 | 0.884 | 0.883 | 0.870 | 0.875 | 0.774 | 0.825 |
| WGS | GBLUP | | | - | 0.785 | 0.760 | 0.839 | 0.828 | 0.883 | 0.881 | 0.811 | 0.852 |
|  | GHBLUP | | | 0.1 | 0.790 | 0.768 | 0.840 | 0.828 | 0.861 | 0.862 | 0.799 | 0.852 |
|  |  |  |  | 0.2 | 0.786 | 0.764 | 0.839 | 0.829 | 0.869 | 0.867 | 0.811 | 0.859 |
|  |  |  |  | 0.3 | 0.786 | 0.762 | 0.838 | 0.827 | 0.871 | 0.868 | 0.811 | 0.857 |
|  |  |  |  | 0.4 | 0.785 | 0.761 | 0.837 | 0.826 | 0.869 | 0.866 | 0.807 | 0.851 |
|  |  |  |  | 0.5 | 0.784 | 0.759 | 0.837 | 0.826 | 0.870 | 0.867 | 0.807 | 0.850 |
|  |  |  |  | 0.6 | 0.782 | 0.758 | 0.837 | 0.827 | 0.872 | 0.869 | 0.805 | 0.848 |
|  |  |  |  | 0.7 | 0.783 | 0.759 | 0.837 | 0.828 | 0.872 | 0.869 | 0.805 | 0.846 |
|  |  |  |  | 0.8 | 0.785 | 0.761 | 0.837 | 0.829 | 0.873 | 0.870 | 0.808 | 0.847 |
|  |  |  |  | 0.9 | 0.783 | 0.760 | 0.838 | 0.829 | 0.874 | 0.871 | 0.807 | 0.847 |
|  | GH+GBLUP | | | 0.1 | 0.779 | 0.763 | 0.830 | 0.822 | 0.854 | 0.857 | 0.816 | 0.865 |
|  |  |  |  | 0.2 | 0.782 | 0.763 | 0.837 | 0.830 | 0.869 | 0.867 | 0.818 | 0.866 |
|  |  |  |  | 0.3 | 0.785 | 0.765 | 0.837 | 0.831 | 0.872 | 0.869 | 0.815 | 0.859 |
|  |  |  |  | 0.4 | 0.754 | 0.763 | 0.836 | 0.829 | 0.866 | 0.862 | 0.809 | 0.851 |
|  |  |  |  | 0.5 | 0.783 | 0.759 | 0.835 | 0.828 | 0.867 | 0.863 | 0.808 | 0.847 |
|  |  |  |  | 0.6 | 0.780 | 0.756 | 0.834 | 0.828 | 0.870 | 0.866 | 0.804 | 0.841 |
|  |  |  |  | 0.7 | 0.780 | 0.757 | 0.833 | 0.830 | 0.867 | 0.864 | 0.802 | 0.839 |
|  |  |  |  | 0.8 | 0.782 | 0.760 | 0.834 | 0.831 | 0.861 | 0.860 | 0.802 | 0.840 |
|  |  |  |  | 0.9 | 0.779 | 0.759 | 0.834 | 0.831 | 0.862 | 0.860 | 0.803 | 0.841 |

Val_LM = validation set from the LM Yorkshire population; Val_XD = validation set from the XD Yorkshire population; Ref_LM = only using the LM Yorkshire population as reference population; Ref_ALL = using the LM and XD Yorkshire population as reference population; Ref_XD = only using the XD Yorkshire population as reference population; NBA = number of piglets born alive; TNB = total number of piglets born; 80K = 80K SNP panel; WGS = imputed whole-genome sequencing data;

^1^The nine different LD thresholds set from $r^{2}\geq0.1$ to $r^{2}\geq0.9$.
